# Silicon Nitride: A Synthetic Mineral for Vertebrate Biology

**DOI:** 10.1038/srep31717

**Published:** 2016-08-19

**Authors:** Giuseppe Pezzotti, Bryan J. McEntire, Ryan Bock, Marco Boffelli, Wenliang Zhu, Eleonora Vitale, Leonardo Puppulin, Tetsuya Adachi, Toshiro Yamamoto, Narisato Kanamura, B. Sonny Bal

**Affiliations:** 1 Ceramic Physics Laboratory, Kyoto Institute of Technology, Sakyo-ku, Matsugasaki, 606-8126 Kyoto, Japan; 2 Amedica Corporation, 1885 West 2100 South, Salt Lake City, 84119 Utah, USA; 3 Department of Medical Engineering for Treatment of Bone and Joint Disorders, Osaka University, 2-2 Yamadaoka, Suita, Osaka 565-0854, Japan; 4 Department of Molecular Cell Physiology, Graduate School of Medical Science, Kyoto Prefectural University of Medicine, 465 Kajii-cho, Kamigyo-ku, Kyoto 602-8566, Japan; 5 Department of Dental Medicine, Graduate School of Medical Science, Kyoto Prefectural University of Medicine, Kamigyo-ku, Kyoto 602-8566, Japan; 6 Department of Orthopaedic Surgery, University of Missouri, Columbia, 65212 Missouri, USA

## Abstract

The remarkable stoichiometric flexibility of hydroxyapatite (HAp) enables the formation of a variety of charged structural sites at the material’s surface which facilitates bone remodeling due to binding of biomolecule moieties in zwitterionic fashion. In this paper, we report for the first time that an optimized biomedical grade silicon nitride (Si_3_N_4_) demonstrated cell adhesion and improved osteoconductivity comparable to highly defective, non-stoichiometric natural hydroxyapatite. Si_3_N_4_’s zwitterionic-like behavior is a function of the dualism between positive and negative charged off-stoichiometric sites (*i.e.*, N-vacancies versus silanols groups, respectively). Lattice defects at the biomaterial’s surface greatly promote interaction with positively- and negatively-charged functional groups in biomolecules, and result in the biologically effective characteristics of silicon nitride. These findings are anticipated to be a starting point for further discoveries of therapeutic bone-graft substitute materials.

The major challenge facing modern orthopaedic devices is their conspicuous inability to adapt or respond to changing physiologic conditions because they are fabricated from non-living bioinert substances. Conversely, *Nature* took a peculiarly different approach when it chose hydroxyapatite (HAp) as its fundamental building material for vertebrates^1^,^2^. While biologic HAp is also non-living, it is not bioinert. Indeed, HAp’s unique crystal structure is capable of being greatly distorted to accommodate a wide range of cation/anion sizes, with up to 20% of these sites substituted with other elemental groups (*e.g*., Na^+^, Mg^2+^, K^+^, Sr^2+^, CO_3_^2−^, HPO_4_^2−^, Cl^−^, F^−^, etc.)^3^. This remarkable stoichiometric flexibility enables the formation of a variety of charged structural sites at the material’s surface which facilitates bone remodeling due to binding of biomolecule moieties in zwitterionic fashion^4^. HAp’s natural surface chemistry is difficult to synthetically replicate. As a result, man-made HAp formulations are suboptimal in terms of osteoconductivity and cell-adhesion^5^. Natural HAp’s unique defect chemistry inspired the current investigation of an alternative synthetic ceramic, silicon nitride (Si_3_N_4_)^6^, to see if it also exhibits zwitterionic-like functionality. Prior studies investigated modifications of its surface chemistry to introduce both positively- and negatively-charged defects, and then classified these sites according to basic concepts of physical chemistry^7^,^8^. In contrast to other more common biomaterials (*i.e*., alumina and Ti-alloys), we report for the first time that an optimized biomedical grade Si_3_N_4_ demonstrated cell adhesion and improved osteoconductivity comparable to highly defective, non-stoichiometric natural HAp. Si_3_N_4_’s zwitterionic-like behavior is a function of the dualism between positive and negative charged off-stoichiometric sites (*i.e.*, N-vacancies versus silanols groups, respectively)^8^, analogous to the way defective Ca^2+^ and PO_4_^3−^ sites perform in HAp^2^. These findings are anticipated to be a starting point for further discovery of therapeutic bone graft substitute materials.

The Si_3_N_4_ of this study (Amedica Corporation, Salt Lake City, UT) contained 6 weight percent (wt.%) yttrium oxide (Y_2_O_3_) and 4 wt.% aluminum oxide (Al_2_O_3_) as densification additives. It was prepared by sintering in nitrogen at a temperature in excess of 1700 °C for ~3 hours, followed by hot-isostatic pressing above 1650 °C under a nitrogen pressure of >200 MPa for ~2 hours. The resulting two-phase microstructure consisted of anisotropic ß-Si_3_N_4_ grains intermixed with a partially crystallized glassy grain-boundary phase of silicon-yttrium-aluminum-oxynitride (Si(Y)AlON)^9^. Preliminary energy dispersive X-ray analyses revealed a composition of the Si(Y)AlON glass 20.8 at.% Si, 20.8 at.% Y, 9.5 at.% Al, 44.3 at.% O, and 4.5 at.% N.

Circular disk samples of this base material (12.7 mm and 1 mm in diameter and thickness, respectively), designated “as sintered” (AS), were subsequently polished and lapped to less than 20 nm Ra surface finish and cleaned in deionized water to remove contaminants. Three separate surface treatments were then performed on these samples including wet-chemical etching in hydrofluoric acid (HF), high-temperature nitrogen atmosphere annealing (NA), and high-temperature thermal oxidation (TO). HF etching of the AS Si_3_N_4_ removed the amphoteric silica (SiO_2_) passivation layer without significantly affecting the underlying nitride grains. This treatment was expected to maximize the concentration of surface amine groups, biasing the composition as far as possible to the nitride end of the nitride-oxide spectrum. The NA treatment (1400 °C for 30 min. under filtered N_2_ at ~115 kPa) was adopted with the intent of increasing the density of surface amines relative to hydroxyl groups. The TO treatment (7 h at 1070 °C in an open-air kiln) was aimed at completely oxidizing the surface, maximizing the concentration of hydroxyl groups and biasing the composition as far as possible to the oxide end of the nitride-oxide spectrum. X-ray photoelectron spectroscopy data, which we extensively presented in a previous paper^7^, showed a surface composition for the NA samples with N/Si and O/Si atomic ratios equal to 1.02 and 0.5, respectively. On the other hand, the TO samples showed N/Si and O/Si atomic ratios equal to 0.09 and 1.98, respectively. Characterizations of the mechanical properties of the surface treated materials are yet ongoing. However, preliminary data showed minimal variation of bulk properties for strength and toughness as compared to the AS sample. Each sample group was then spectroscopically examined and subsequently subjected to biologic testing and characterization.

The Raman spectra (RS) were recorded in backscattering using a triple monochromator (T-64000, Jobin-Yvon, Horiba Group, Kyoto, Japan) and an excitation source at 532 nm. Cathodoluminescence (CL) spectra were collected in a field-emission gun scanning electron microscope (FEG-SEM, SE-4300, Hitachi Co., Tokyo, Japan). For all samples, exactly the same experimental conditions were applied (acceleration voltage and beam current fixed at 6 kV and 180 pA, respectively). The electron-stimulated luminescence was analyzed by a high spectrally resolved monochromator (Triax 320, Jobin-Yvon, Horiba Group, Tokyo, Japan).

Cell proliferation and osteoconductivity were assessed using human osteosarcoma cells (SaOS-2) with a density of 5 × 10^5^ cell/ml seeded onto the treated Si_3_N_4_ disks within petri-dishes. In the cell-proliferation experiments, cells were incubated in 4.5 g/L glucose DMEM (D-glucose, L-Glutamine, Phenol Red, and Sodium Pyruvate) supplemented with 10% fetal bovine serum and allowed to proliferate within each petri-dish for about 24 h at 37 °C. The proliferation time was selected according to a previously published study by other authors^10^. Subsequently, the cells were stained for fluorescence microscopy with Phalloidin (green; F-actin) and Hoechst 33342 (blue; nuclei) for 1 hour, and washed three times with 1 mL TBST solution.

Cell counts were then performed on more than 30 fluorescence micrographs for each of the samples. sRANKL and OPG were quantified in cell conditioned media using the R&D System ELISA kits, MTR00 and MOP00, respectively, according to the manufacturer’s instructions. Titration of free sRANKL was computed by the difference between the equivalent weight of sRANKL and OPG obtained from ELISA, assuming 1:1 as reactive normality of sRANKL:OPG ratio. Human recombinant sRANKL (Peprotech, Cat. #310-01) was used for the *in vitro* osteoclastogenic assay^11^. In the osteoconductivity tests, cell seeding took place in an osteogenic medium which consisted of DMEM supplemented with about 50 μg/mL ascorbic acid, about 10 mM β-glycerol phosphate, 100 mM hydrocortisone, and about 10% fetal bovine calf serum. The samples were incubated for 7 days at 37 °C. Results were assessed by laser microscopy and 3D image analyses. All experiments were repeated in triplicate, with data expressed as means ± one standard deviation. Statistical analysis was performed according to the unpaired Student’s t-test or to one-way Analysis of Variance (ANOVA). A *p* value < 0.05 was considered statistically significant.

Figure 1(a–c) are fluorescence micrographs of SaOS-2 cells after 24 h proliferation on the NA, TO, and AS Si_3_N_4_ samples, respectively. Upon visual inspection, the NA sample showed the highest affinity for cell adhesion and proliferation among the series of tested Si_3_N_4_ samples. Looking at the CL spectra from differently treated Si_3_N_4_ materials in (d), its biological affinity was found to be directly proportional to the population of positively charged defects (*i.e*., nitrogen vacancies and N-N bonds; *cf*. labels). Figure 2(a–c) show three-dimensional laser micrographs of the HAp structure developed on the NA, TO, and AS Si_3_N_4_ samples after one week’s activity of the SaOS-2 cells, respectively. The NA sample showed the most significant HAp growth, with locations as high as 0.4 ~ 0.5 mm. In (d), a summary is shown of the Raman characterization of the surfaces of these various samples. These analyses confirmed both the formation of HAp (*cf*. PO_4_^3−^stretching at ~960 cm^−1^, and labels) and a higher average amount of HAp on the NA samples. Results from the statistical evaluation of cell proliferation and HAp formation for all Si_3_N_4_ samples in comparison with commercially available alumina and Ti-alloy biomaterials are given in Fig. 3(a,b), respectively. Again, the highest activity was found on the NA Si_3_N_4_ samples. Their improvement was significant in terms of cell proliferation (*cf*. plot of number of cells per unit area in Fig. 3(a)) and HAp growth (*cf*. plot of formed HAp volume per unit area in Fig. 3(b) as obtained by three-dimensional laser microscopy), not only with respect to the AS Si_3_N_4_ samples, but also in comparison to the alumina and Ti-alloy biomaterials. Photographs of water contact angles, shown at the top of Fig. 3, demonstrate that the improved hydrophilicity of the tested samples matches their biological affinity. Thermal treatments of the AS Si_3_N_4_ samples produced extremely low contact angles (*cf*. Fig. 3), measured using a goniometer technique, as previously reported^7^. The affinity of the various Si_3_N_4_ samples for osteoblast cell attachment in comparison with other biomaterials was confirmed by results from the sRANKL experiments shown in Fig. 3(c). The observed deficiency in the Receptor Activator of NF-kB Ligand (RANKL), a membrane-bound protein cleaved into soluble sRANKL by metalloproteinase 14, revealed a low propensity for osteoclast formation, suggesting that Si_3_N_4_ presents a biologically friendly surface. Considering the physical chemistry characteristics of the Si_3_N_4_ surface and its favorable cell adhesion/osteoconduction performance, it is hypothesized that the thermal treatments produce a mixture of negatively- and positively-charged functional surface groups (*i.e*., silanols, silicon-amines, and N-vacancies, *etc*.) that are capable of binding biomolecules in zwitterionic fashion.

While streaming potential measurements yield a net negative surface charge at homeostatic pH^7^, positive charges are also present. Besides amine groups, Y-OH and Al-OH groups (*i.e*., from sintering additives) likely protonate and become positively charged at physiologic pH. They can substitute for silanols, thereby increasing the zwitterionic-like character of the surface. Another significant finding was the formation of a peculiar Si(Y)AlON phase on the surface of the NA sample, which increased the positively charged defective sites as observed by CL. These defects intimately mix at the atomic level with the silanol-dominated (*i.e*., negatively-charged) Si_3_N_4_ surface. Due to their predominantly positive charge, N-vacancies and defects associated with N-N bonds further the zwitterionic-like character of the surface. A significant amount of differential surface charge is therefore present on Si_3_N_4_ in comparison to oxide biomaterials (*e.g*., alumina, which exhibits only one dominant functional group). Evidence for this assertion is the striking similarity found for the distribution and morphology of the newly formed Si(Y)AlON phase and the proliferated cells on the surface of the NA Si_3_N_4_ sample (*cf*. Fig. 4). The morphology of the Si(Y)AlON phase formed on the surface, which is shown in the laser micrograph in Fig. 4(a), resembled the location of proliferating cells on the surface of the NA Si_3_N_4_ sample after 24 h of SaOS-2 proliferation (given in the scanning electron micrograph on fixed sample in Fig. 4(b)).

A significant amount of interest has been generated over the past decade in functionalizing biomaterial surfaces to be zwitterionic for anti-fouling purposes^12^. A surface where positive and negative charges are intimately mixed allows for extreme hydrophilicity (as observed on the current surface-treated Si_3_N_4_ materials); it also prevents bacterial adhesion (as previously reported for AS Si_3_N_4_ samples)^13^. Moreover, zwitterated surfaces have also been observed to induce greater apatite formation on titanium alloys^14^. The zwitterionic-like properties of the NA Si_3_N_4_ samples are derived from: (i) a significant but minority population of amine sites that are positively-charged; (ii) substitutional Y and Al on Si sites yielding positively-charged Y-OH_2_^+^ and Al-OH_2_^+^ groups; (iii) an increased concentration of positively-charged lattice defects; and (iv) an intermixed layer of negatively-charged Si-O^−^ groups. As more positive charges become admixed into a mostly negative surface, it exhibits greater zwitterionic, hydrophilic, and osteopromotive characteristics. Consequently, lattice defects in the biomaterial’s surface greatly promote interaction with positively and negatively charged functional groups in biomolecules. Further studies, presently ongoing, seem also to confirm a favored osteoblast differentiation for the NA sample above other surface treatments. Similar to naturally occurring HAp, Si_3_N_4_ illustrates the concept that atomically defective materials exhibit biologically effective characteristics.

## Methods

### Cell proliferation experiments

The SaOS-2 cells used in this research were cultured in DMEM medium consisting of 4.5 g/L D-glucose, L-Glutamine, Phenol Red and Sodium Pyruvate, in addition to 10% of fetal bovine serum. Cell proliferation and osteoconductivity were assessed using an initial cell density of 5 × 10^5^ cell/ml seeded onto the treated Si_3_N_4_ disks within conventional petri dishes. Cells were allowed to proliferate within the petri dish for 24 h at 37 °C. The cells were then fixed with 4% paraformaldehyde (PFA) for 10 min and permeabilized in TBS-0.1% Triton X for 5 min. Before each of these two steps, the cells were washed with 1 mL TBS (20 mM Tris-HCl pH 7.5 and 150 mM NaCl). Finally, the cells were washed with 1 mL TBST (TBS, 0.05% tween 20 and 0.05% NaN_3_) for 1 hour.

For visualization, the cells were stained for fluorescence microscopy with Phalloidin (green; F-actin) and Hoechst 33342 (blue; nuclei) for 1 hour, and washed three times with 1 mL TBST solution (mixture of Tris-Buffered Saline and Tween 20).

### Osteoconductivity experiments

In osteoconductivity tests, cell seeding took place in an osteogenic medium, which consisted of DMEM supplemented with about 50 μg/mL ascorbic acid, about 10 mM β-glycerol phosphate, 100 mM hydrocortisone, and about 10% fetal bovine calf serum. The samples were incubated for 7 days at 37 °C. Results were assessed by laser microscopy and 3D image analyses, as explained later.

### Raman experiments

The Raman spectra were recorded in backscattering using a triple monochromator (T-64000, Jobin-Yvon, Horiba Group, Kyoto, Japan) equipped with liquid nitrogen-cooled charge coupled device (CCD), a confocal pinhole, and polarization filters. The excitation source in the present experiments used a 532 nm Nd:YVO_4_ diode-pumped solid-state laser (SOC JUNO, Showa Optronics Co. Ltd., Tokyo, Japan) operating with a power of 200 mW. An objective lens with a numerical aperture of 0.5 was used both to focus the laser beam on the sample surface and to collect the scattered Raman light. Confocal experiments were conducted with a pinhole aperture of 100 μm and by employing an objective lens with a magnification of 100x. Spectral lines were analyzed with the aid of a commercially available software package (Labspec 4.02, Horiba/Jobin-Yvon, Kyoto-Japan).

### Cathodoluminescence experiments

Cathodoluminescence spectra were collected in a field-emission gun scanning electron microscope (FEG-SEM, SE-4300, Hitachi Co., Tokyo, Japan). All samples were analyzed during the same experimental session, with exactly the same experimental conditions being applied (acceleration voltage and beam current fixed at 6 kV and 180 pA, respectively). The nominal spatial resolution of the electron beam at the sample surface was 1.5 nm. The microscope was equipped with a CL device consisting of an ellipsoidal mirror, and a bundle of optical fibers, used to collect and to focus, respectively, the electron-stimulated luminescence emitted by the sample into a high spectrally resolved monochromator (Triax 320, Jobin-Yvon/Horiba Group, Tokyo, Japan). The obtained spectra were deconvoluted into Gaussian sub-bands using commercially available software (Origin 9.1, OriginLab Co., Northampton, MA, USA).

### Laser microscopy experiments

The optical morphologies of the samples were characterized in a back-scattered confocal laser microscope (Keyence, VK-X210, Osaka, Japan). The excitation source in the experiments used a 408 nm violet semiconductor laser operating with an output of 0.95 mW. An objective lens with a numerical aperture of 0.55 was used both to focus the laser beam on the sample surface and to collect the reflected light. Taking advantage of an automated stage, whose movement can be controlled in xyz directions, a 3 dimensional colored image of the sample surface can be acquired by joining the 3D profile with the optical microscope image.

## Additional Information

**How to cite this article**: Pezzotti, G. *et al*. Silicon Nitride: A Synthetic Mineral for Vertebrate Biology. *Sci. Rep.* **6**, 31717; doi: 10.1038/srep31717 (2016).

## Figures and Tables

**Figure 1:**
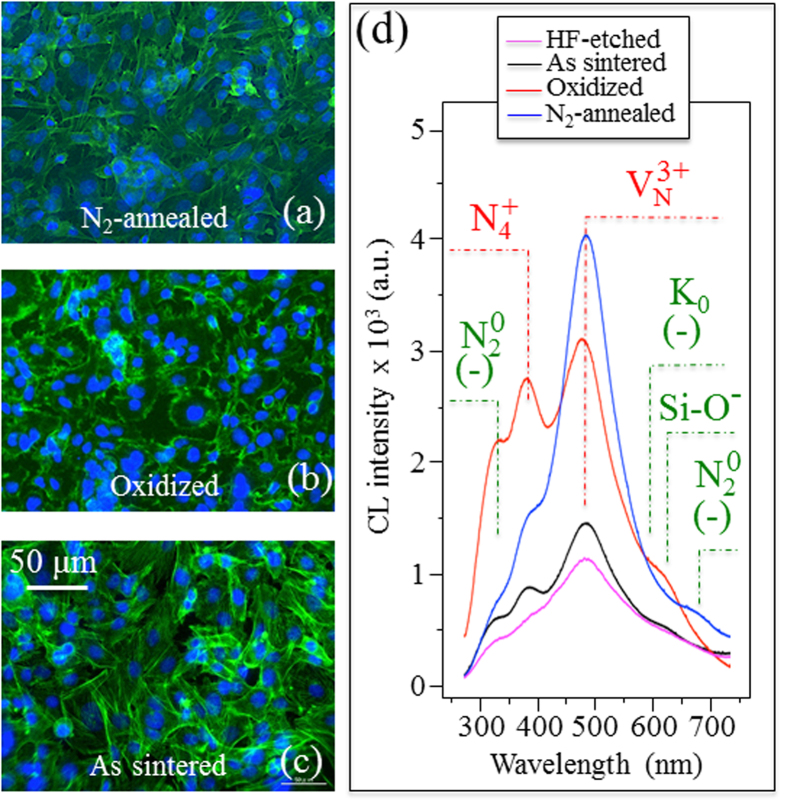
SaOS-2 cell proliferation after 24 h as visualized by fluorescence spectroscopy on the surface of NA (**a**), TO (**b**), and AS (**c**) Si_3_N_4_ samples. In (**d**), CL spectra are plotted for all investigated Si_3_N_4_ samples. The labels N_2_^0^, N_4_^+^, V_N_^3+^, K_0_, Si-O^−^ and N_2_^0^ refer to N dangling bonds, N-N bonds, N vacancies, Si dangling bonds, non-bridging oxygen hole centers in surface SiO_2_, and N dangling bonds, respectively^7^. The minus in brackets labels the defects with a negative charge.

**Figure 2:**
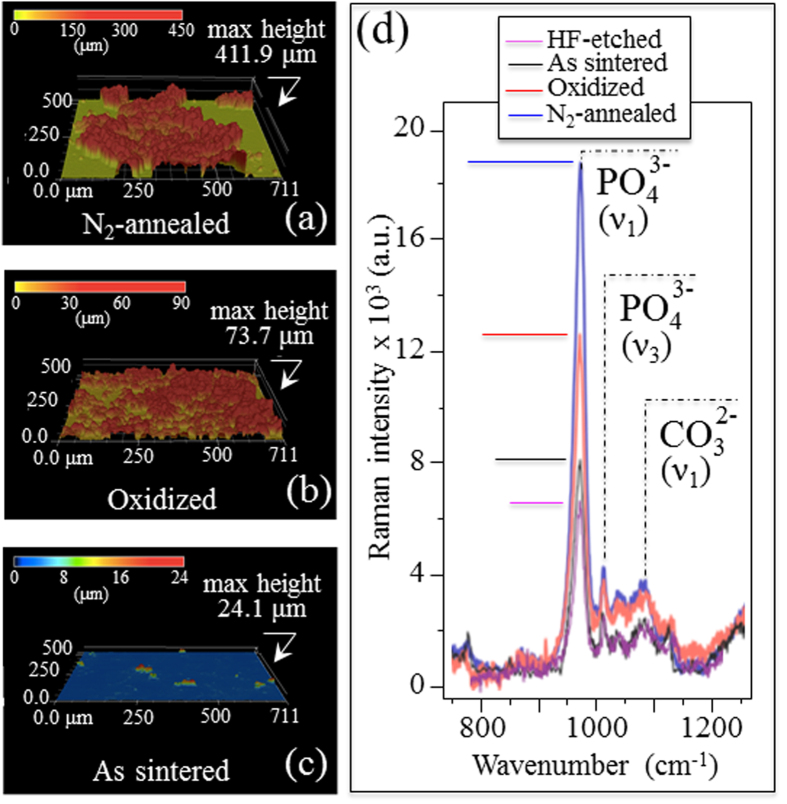
Three-dimensional laser micrographs of HAp grown on the surface of NA (**a**), TO (**b**), and AS (**c**) Si_3_N_4_ samples after 1-week activity of SaOS-2 cells. In (**d**), Raman spectra are plotted for all investigated Si_3_N_4_ samples. The labels PO_4_^3−^ (*ν*_1_) and PO_4_^3−^ (*ν*_3_) refer to symmetric and asymmetric stretching vibrations of the P-O bonds of hydroxyapatite, respectively, while CO_3_^2−^ (*ν*_1_) refers to the symmetric stretching mode of the substitutional carbonate. The intermediate band between the latter two (not labeled) represents out-of-plane asymmetric stretching of the PO_4_^3−^ tetrahedra.

**Figure 3:**
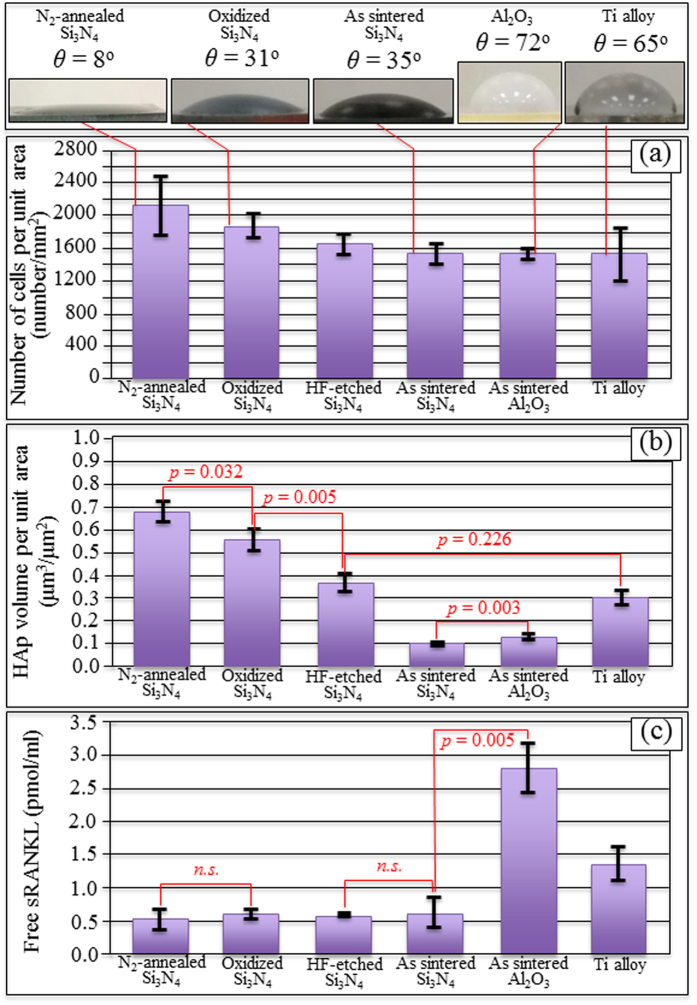
Results of a statistical evaluation of cell proliferation (**a**) and HAp formation (**b**) for all Si_3_N_4_ samples in comparison with commercially available alumina and Ti alloy biomaterials tested under exactly the same conditions. In (**c**), affinity for osteoblast cell attachment for samples is assessed by sRANKL experiments. On top, photographs and values of sessile (water) wetting angle, *θ*, are shown for different materials (i.e., as measured at equilibrium after 30 min from drop deposition).

**Figure 4:**
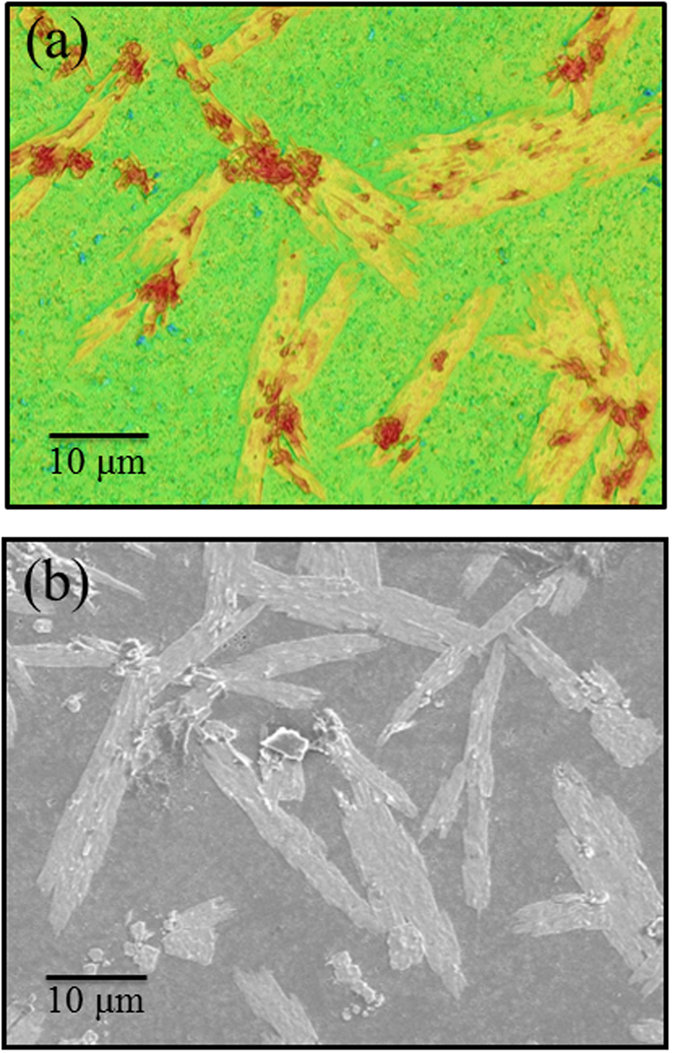
Similarity found between the morphology of the newly formed Si(Y)AlON phase (laser micrograph in (**a**)) and the location of proliferating cells on the surface of the NA Si_3_N_4_ sample after 24 h of SaOS-2 proliferation (scanning electron micrograph on fixed sample in (**b**)).
